# 钴镍笼状双金属氢氧化物/多壁碳纳米管复合材料对环境水样中农药的高效富集

**DOI:** 10.3724/SP.J.1123.2022.03011

**Published:** 2022-10-08

**Authors:** Xuemei WANG, Jing YANG, Jiali ZHAO, Zheng ZHOU, Xinzhen DU, Xiaoquan LU

**Affiliations:** 西北师范大学化学化工学院, 甘肃省生物电化学与环境分析重点实验室, 甘肃 兰州 730070; Key Laboratory of Bioelectrochemistry and Environmental Analysis of Gansu Province, College of Chemistry and Chemical Engineering, Northwest Normal University, Lanzhou 730070, China

**Keywords:** 钴镍双金属氢氧化物, 多壁碳纳米管, 固相微萃取, 高效液相色谱, 农药, cobalt-nickel layered double hydroxide (CoNi-LDH), multi-walled carbon nanotubes (MWCNTs), solid-phase microextraction (SPME), high performance liquid chromatography (HPLC), pesticides

## Abstract

建立高效、灵敏的农药分离、富集和检测方法具有重要意义。该实验采用一步法合成了钴基沸石咪唑骨架/多壁碳纳米管(ZIF-67/MWCNTs)复合物,并以该复合物为模板通过溶剂热法合成了钴镍笼状双金属氢氧化物/多壁碳纳米管(CoNi-LDH/MWCNTs)复合材料,将CoNi-LDH/MWCNTs用作固相微萃取(SPME)的纤维涂层富集环境水样中的6种农药,结合高效液相色谱(HPLC)测定了环境水样中的6种农药。通过扫描电镜、能谱分析、红外光谱、粉末X射线衍射和N_2_吸附/脱附对所制备的各种材料进行了表征。利用正交设计试验优化SPME的萃取条件,包括萃取温度、萃取时间、搅拌速率、解吸时间和盐浓度。在最优化的条件下,该方法具有较宽的线性范围(百菌清为0.015~200 μg/L,戊唑醇为0.140~200 μg/L,毒死蜱为0.250~200 μg/L,仲丁灵为0.077~200 μg/L,溴氰菊酯为1.445~200 μg/L,哒螨灵为0.964~200 μg/L)、较低的检出限(0.004~0.434 μg/L)和良好的重复性。单个纤维和不同批次纤维间的相对标准偏差(RSD)分别为0.5%~5.7%和0.5%~4.8%。在10.0 μg/L和50.0 μg/L 2个水平下的加标回收率为83.9%~108.2%, RSD< 5.3%。此外,与其他涂层纤维相比,CoNi-LDH/MWCNTs涂层对农药具有更高效的富集能力,这归因于它的高比表面积以及CoNi-LDH/MWCNTs涂层与目标分析物之间存在的*π*-*π*堆积作用、疏水作用、阳离子-*π*相互作用和氢键作用。该方法可以实现环境水样中农药残留的高选择性、高灵敏度及高准确性的分析测定。

## Abstract (en)

Pesticides are widely used in agriculture to increase grain yields and prevent crop diseases and insect pests. However, pesticides pose a serious threat to ecosystems and human health owing to their high toxicity and persistence. Therefore, it is imperative to establish an efficient and sensitive detection method for pesticides in water samples. Rapid and accurate detection of trace pesticides in environmental water samples has been a challenge because of complex matrix effects and trace concentrations. Appropriate sample pretreatment is a critical step for the effective extraction of analytes and removal of interferences, and the development and design of novel and stable nanomaterial adsorbents is key to continuous innovation in sample pretreatment technology. In recent years, carboxylated multiwalled carbon nanotubes (MWCNTs-COOH) and layered double hydroxide (LDHs) have been widely used as new adsorbent materials for various pretreatment technologies because of their large specific surface area, good stability, and easy functionalization. Based on this background, MWCNTs-COOH and LDHs were combined to obtain a new efficient composite adsorbent, so that the synergistic effect of the individual components could be exploited in entirety. In this study, a zeolitic metal organic framework ZIF-67/multiwalled carbon nanotube (ZIF-67/MWCNTs) composite was prepared by a simple one-step method, and a cobalt-nickel double metal hydroxide/multiwalled carbon nanotube (CoNi-LDH/MWCNTs) hybrid material with a three-dimensional cage-like structure was synthesized by a solvothermal method using ZIF-67/MWCNTs as templates. The cage-like structure of the CoNi-LDH/MWCNTs composite, which is different from the traditional layered bimetallic hydroxide, could accelerate mass transfer. Given the excellent properties of the CoNi-LDH/MWCNTs composite, it was used as a solid-phase microextraction (SPME) coating for the efficient enrichment of six pesticides (chlorothalonil, tebuconazole, chlorpyrifos, butralin, deltamethrin, and pyridaben) and combined with high performance liquid chromatography-ultraviolet (HPLC-UV) detection for the determination of the six pesticides in real water samples. The prepared materials were characterized by scanning electron microscopy, electron dispersion spectroscopy, infrared spectroscopy, X-ray powder diffraction, and N_2_ adsorption/desorption. The results confirmed that the CoNi-LDH/MWCNTs composite was successfully synthesized, and that its surface area and pore volume were 281.4 m^2^/g and 0.49 cm^3^/g, respectively. An orthogonal array design was used to optimize the extraction conditions of SPME, including the extraction time, extraction temperature, stirring rate, salt effect, and desorption time. The optimal extraction conditions were as follows: extraction temperature, 40 ℃; extraction time, 30 min; stirring rate, 500 r/min; desorption time, 6 min; and salt (NaCl) mass concentration, 150 mg/L.

Under optimal conditions, the method had a wide linear range (chlorothalonil: 0.015-200 μg/L, tebuconazole: 0.140-200 μg/L, chlorpyrifos: 0.250-200 μg/L, butralin: 0.077-200 μg/L, deltamethrin: 1.445-200 μg/L, pyridaben: 0.964-200 μg/L), low detection limit (0.004-0.434 μg/L), and good reproducibility. The relative standard deviations (RSDs) of single fiber and fiber-to-fiber were in the range of 0.5% to 5.7% and 0.5% to 4.8%, respectively. The spiked recoveries at two levels of 10.0 μg/L and 50.0 μg/L were in the range of 83.9%-108.2%, with RSDs less than 5.3%. Compared with other coated fibers (MWCNTs-COOH, ZIF-67, ZIF-67/MWCNTs, and silicone sealant), the CoNi-LDH/MWCNTs-coated fibers showed a better enrichment effect for pesticides, which was attributed to their high specific surface area and *π*-*π* interactions, hydrophobic interactions, cation-*π* interactions, and hydrogen bonding interactions between the CoNi-LDH/MWCNTs coating and the target analytes, which can enhance their ability to extract pesticides. The stability test on the SPME fibers revealed that after 128 cycles, the extraction efficiency of the CoNi-LDH/MWCNTs-coated fibers for the six pesticides decreased only slightly (< 10%), implying that the coated fibers had good stability and reusability. Therefore, this method can be used to detect pesticide residues in environmental water samples with high selectivity, sensitivity, and accuracy.

为了提高粮食产量和防止作物虫害,农药被广泛地应用于农业中,而农药的过度和不当使用是造成水污染的一个主要来源。据统计,使用的农药只有1%进入了目标生物体内,而其余的通过大气干、湿沉降等作用直接或间接地进入土壤、水体和大气中^[1]^。由于农药具有高毒性和持久性,对生态系统和人类健康构成了严重威胁。有文献报道,水体中的农药易于在生物体中积累,通过食物链增加人类中毒和疾病的风险,并可能导致如癌症、不孕、畸形以及由此引起的染色体变化、DNA突变等危害^[2⇓-4]^。因此,开发一种简便、安全、快速和灵敏的分析方法吸附和去除水环境中的农药具有重要的意义。

目前,已经报道了许多分析方法检测环境水体中的农药,主要包括气相色谱法(GC)、气相色谱-质谱法(GC-MS)、离子迁移谱法(IMS)、高效液相色谱法(HPLC)等^[5⇓⇓⇓⇓-10]^。由于环境水样基质复杂、成分多样以及农药的痕量水平,仪器无法直接检测到水中超痕量的农药^[11]^。因此在进行仪器分析之前,样品的富集、浓缩和预处理是必要过程。最常用的预富集方法主要有固相萃取(SPE)、固相微萃取(SPME)、分散液-液微萃取(DLLME)、磁性固相萃取(MSPE)等^[12⇓⇓-15]^。SPME由于具有无溶剂、分离速度快、操作简单和萃取效率高等特点,已被广泛应用于生物、环境、制药和现场分析等领域^[16,17]^。研究表明,纤维涂层的结构和性能对萃取效率和选择性有着至关重要的影响^[18]^。然而,商用SPME纤维成本高、寿命短、选择性较差,为了克服这些缺点,越来越多的研究工作集中在SPME纤维涂层材料的开发上。

层状双金属氢氧化物(LDHs)是一类由带正电荷的片层结构的水镁石和其层间填充带负电荷的阴离子所构成的无机纳米材料^[19]^。由于具有层状结构、高孔隙率和高比表面积等优点,LDHs作为吸附剂具有广阔的应用前景。然而,这类无机吸附剂对疏水性的有机污染物展现出较弱的吸附能力。为了解决该问题,将LDHs与有机纳米材料杂化形成有机-无机复合型吸附剂来提高其对有机污染物的吸附性^[20]^。羧基修饰的多壁碳纳米管(MWCNTs-COOH)因具有较大的比表面积、良好的化学稳定性以及强疏水性而成为一种优异的杂化材料^[21]^。此外,MWCNTs-COOH表面和边缘上含有的大量含氧官能团不仅有助于MWCNTs在水中分散,还提供了与有机污染物形成氢键和静电作用的可能。因此,将MWCNTs-COOH和LDHs制备成复合材料,充分发挥二者协同作用,有望得到一种高效的新型吸附材料。

本研究首先在室温下通过一步法合成了钴基沸石咪唑骨架/多壁碳纳米管(ZIF-67/MWCNTs)复合物,并以该复合物为模板通过溶剂热法合成了钴镍笼状双金属氢氧化物/多壁碳纳米管(CoNi-LDH/MWCNTs)复合材料,将MWCNTs表面生长的ZIF-67作为钴源和模板制备出具有三维笼状结构的CoNi-LDH,有效地防止了LDHs在MWCNTs上的聚集和堆积,从而增强了LDHs的富集效率和吸附能力。最后以不锈钢丝为基底制得CoNi-LDH/MWCNTs涂层的SPME纤维,并结合HPLC-UV测定环境水样中的6种农药(见图1)。

**图1:**
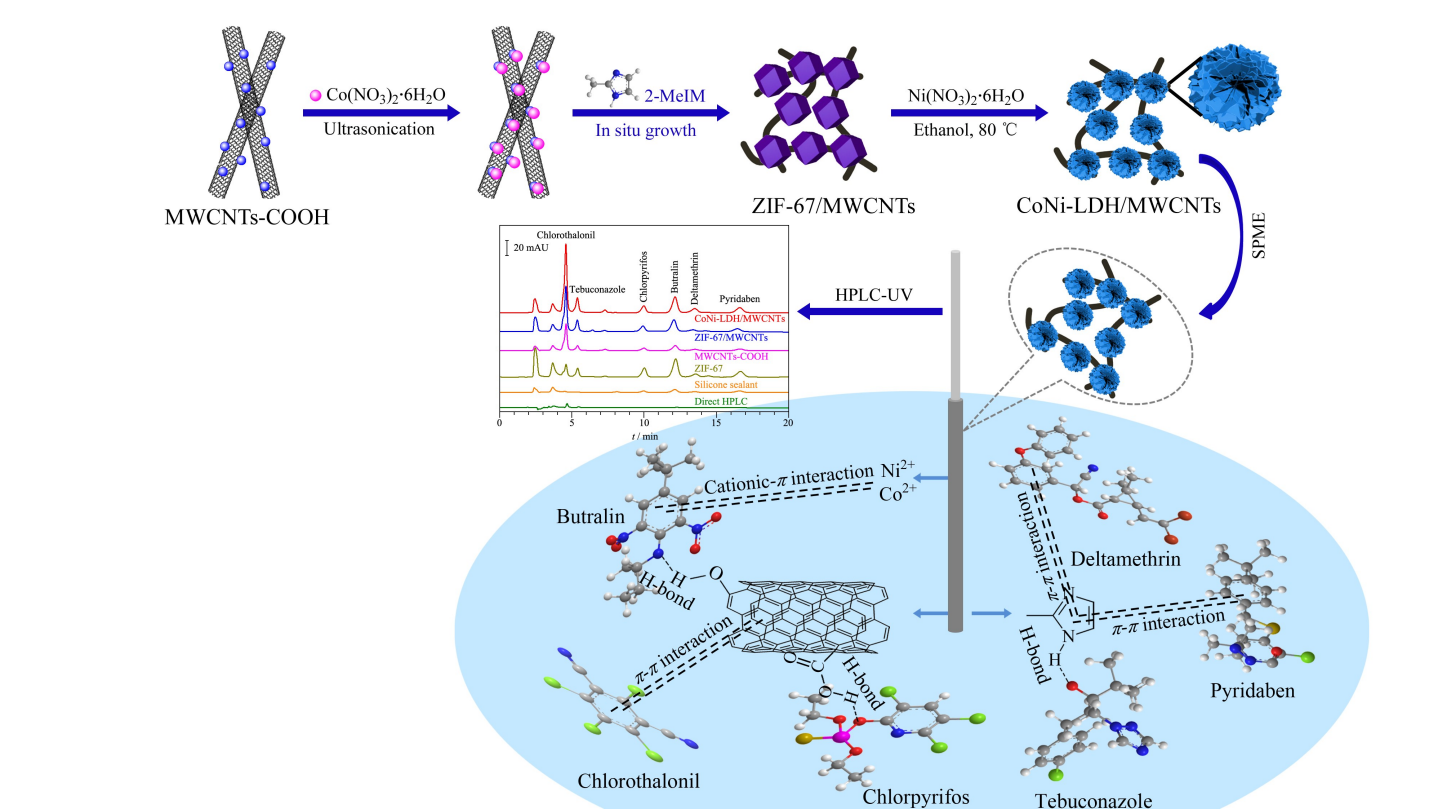
CoNi-LDH/MWCNTs涂层的SPME纤维的制备及其从环境水样中吸附农药的机理

## 1 实验部分

### 1.1 仪器与试剂

Agilent 1260高效液相色谱(美国安捷伦科技有限公司); Ultra Plus热场发射扫描电镜(德国Zeiss公司); FTS-3000傅里叶变换红外光谱仪(美国DIGIL-B公司); D/max-2400粉末X射线衍射仪(日本理学公司); AS1Win比表面积分析仪(美国康塔仪器公司); B11-2恒温磁力搅拌器(上海司乐仪器有限公司); TP-214电子分析天平(丹佛仪器(北京)有限公司); KQ3200E超声波清洗器(昆山市超声仪器有限公司); DZF-6020真空干燥箱(上海一恒科学仪器有限公司); WHF-0.5聚四氟乙烯高压反应釜(北京反应釜有限公司)。

功能化多壁碳纳米管MWCNTs-COOH(纯度>98%,长度<10 μm,直径30~50 nm,中国科学院成都有机化学有限公司);六水合硝酸钴和六水合硝酸镍(分析纯,国药集团化学试剂有限公司); 2-甲基咪唑(分析纯,阿拉丁试剂有限公司);氯化钠(分析纯,天津市凯通化学试剂有限公司);甲苯(分析纯,成都市科隆化学品有限公司);甲醇和乙醇(分析纯,利安隆博华(天津)医药化学有限公司);甲醇和乙腈(色谱纯,赛默飞世尔(上海)有限公司);硅酮密封胶(安徽神舟飞船胶业有限公司);硝酸(分析纯,昆山金城试剂有限公司);盐酸(分析纯,国药集团化学试剂有限公司);不锈钢丝(直径0.3 mm,上海高歌工贸有限公司);实验用水均为Aquapro净水系统制得的超纯水。

6种农药标准品购自国家农药质检中心,分别为百菌清(chlorothalonil,纯度99.8%)、戊唑醇(tebuconazole,纯度99.8%)、毒死蜱(chlorpyrifos,纯度99.5%)、仲丁灵(butralin,纯度99.8%)、溴氰菊酯(deltamethrin,纯度99.8%)、哒螨灵(pyridaben,纯度99.0%),它们的基本信息及化学结构及见表1。

**表1:** 6种农药的基本信息及化学结构

Classification	Chemical	CAS No.		M_r_		Structure	
Fungicides	chlorothalonil (百菌清)	1897-45-6		265.9		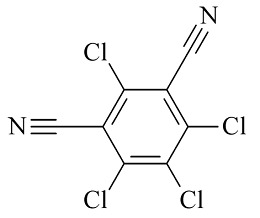	
Fungicides	tebuconazole (戊唑醇)	107534-96-3		307.8		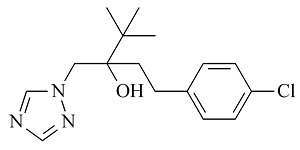	
Acaricide	chlorpyrifos (毒死蜱)	2921-88-2		350.6		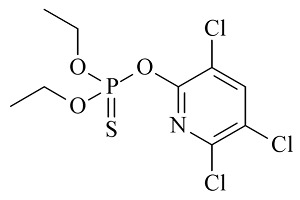	
Herbicide	butralin (仲丁灵)	33629-47-9		295.3		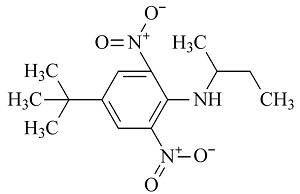	
Insecticide	deltamethrin (溴氰菊酯)	52918-63-5		505.2		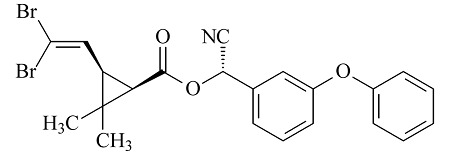	
Acaricide	pyridaben (哒螨灵)	96489-71-3		364.9		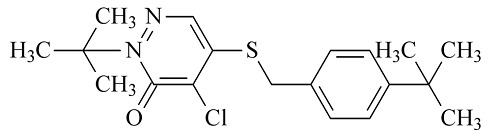	

### 1.2 标准溶液的配制

混合标准储备液:分别准确称取6种标准物质各1 mg,用甲醇配制成10 mg/L的混合标准储备液;标准工作液:用超纯水将混合标准储备液稀释成不同浓度的标准工作液并于4 ℃下避光保存。

### 1.3 实际样品处理

3种实际水样分别采自民勤河(甘肃省白银市)上游、下游以及东大沟(甘肃省白银市)。水样在使用前均用真空抽滤去除不溶性杂质,之后用0.45 μm滤膜过滤,并在4 ℃下储存在棕色玻璃瓶中。

### 1.4 CoNi-LDH/MWCNTs纤维涂层的制备

#### 1.4.1 ZIF-67/MWCNTs复合材料的制备

ZIF-67/MWCNTs根据文献^[22]^报道的方法并加以改进制备得到。首先,称取50 mg的MWCNTs-COOH超声分散于25 mL甲醇中,再加入0.722 g(2.48 mmol)的Co(NO_3_)·6H_2_O超声1 h后形成溶液A;将1.629 g (19.84 mmol)的2-甲基咪唑溶解于25 mL甲醇中形成溶液B;然后将溶液B迅速倒入溶液A中并在室温下搅拌6 h。最后,将得到的黑色产物(ZIF-67/MWCNTs)用甲醇洗涤3次并于60 ℃下真空干燥过夜。

#### 1.4.2 CoNi-LDH/MWCNTs复合材料的制备

称取50 mg的ZIF-67/MWCNTs置于20 mL无水乙醇中并超声分散10 min,再将75 mg的Ni(NO_3_)·6H_2_O溶解于5 mL无水乙醇中并迅速倒入前液,磁力搅拌5 min后转移至高温反应釜中,80 ℃反应2 h,反应完成后通过离心收集产物。最后用无水乙醇洗涤3次,在60 ℃下真空干燥过夜。

#### 1.4.3 CoNi-LDH/MWCNTs涂层纤维的制备

本实验采用直径0.3 mm、长度5 cm的不锈钢丝作为SPME纤维基体。首先将中性硅密封胶用甲苯(300 mg/mL)在离心管中稀释,再把经过酸处理的不锈钢丝浸在硅胶密封胶稀释液中(深度2.5 cm),然后立即拔出并插入CoNi-LDH/MWCNTs粉末中,缓慢取出得到均匀的涂层,相同的过程重复3~4次,可以获得相应厚度的涂层,最后,将获得的纤维浸入到纯硅胶密封胶稀释液中并在其表面形成一层保护膜,随后将制备好的SPME纤维在100 ℃下固化2 h并在室温下保存备用。

### 1.5 SPME过程与液相色谱条件

将制备的CoNi-LDH/MWCNTs纤维浸入装有15 mL水样或标准溶液(含150 mg/L NaCl)的萃取瓶中并在40 ℃、500 r/min的条件下萃取30 min。待萃取完成后,将纤维取出并置于含有200 μL乙腈的解吸管中静态解吸6 min。最后将20 μL解吸液注入HPLC中进行分析。为消除可能的萃取残留物对实验结果的干扰,每次在萃取前依次用乙腈和水清洗CoNi-LDH/MWCNTs纤维20 min和10 min。

使用反相C_18_ HPLC色谱柱(150 mm×4.6 mm, 5 μm,美国安捷伦科技有限公司)进行定量分析。流动相为甲醇-水(85∶15, v/v),流速为0.6 mL/min,紫外检测波长为225 nm,进样量为20 μL,柱温保持在25 ℃。

## 2 结果与讨论

### 2.1 材料的表征

#### 2.1.1 扫描电镜(SEM)

通过SEM对材料的形貌结构进行了探究。如图2a所示,MWCNTs-COOH的直径在30~50 nm之间且形貌为管状结构。图2b和图2c分别是ZIF-67和ZIF-67/MWCNTs的SEM图,结果显示ZIF-67具有规则的十二面体结构且ZIF-67与MWCNTs杂化后,其形貌并未改变。通过图2d可以观察到在MWCNTs上的ZIF-67被成功地转变成具有三维笼状结构的CoNi-LDH,其形成过程可以用柯肯达尔效应和酸性刻蚀来解释。首先,溶液中的Ni^2+^发生水解反应,产生的质子进入ZIF-67骨架的内部,破坏Co^2+^和2-甲基咪唑所形成的配位键并释放出Co^2+^,由于柯肯达尔效应,Co^2+^由内向外迁移且与Ni^2+^共同在ZIF-67的表面形成CoNi-LDH纳米片,最后呈现出三维笼状的结构。图2e和2f表明CoNi-LDH/MWCNTs材料被均匀地涂敷在不锈钢丝上并通过计算得出纤维的涂层厚度约为21.4 μm。

**图2:**
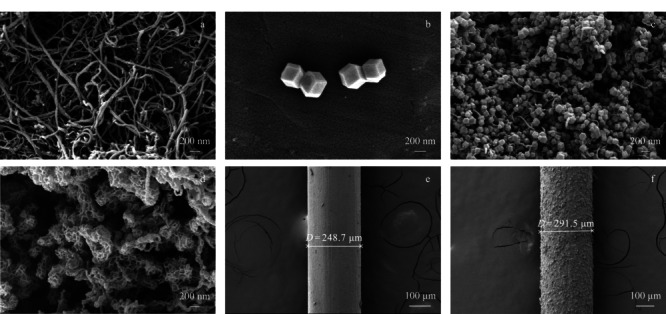
(a)MWCNTs-COOH、(b)ZIF-67、(c)ZIF-67/MWCNTs、(d)CoNi-LDH/MWCNTs、(e)经酸处理的不锈钢丝和(f)CoNi-LDH/MWCNTs纤维的SEM图

#### 2.1.2 能谱(EDS)分析

采用EDS对ZIF-67/MWCNTs和CoNi-LDH/MWCNTs进行了分析。通过图3a和3b可以观察到在图3b的EDS光谱中出现了新的元素Ni,这进一步验证了SEM的分析结果,表明成功地制备了CoNi-LDH/MWCNTs复合材料。

**图3:**
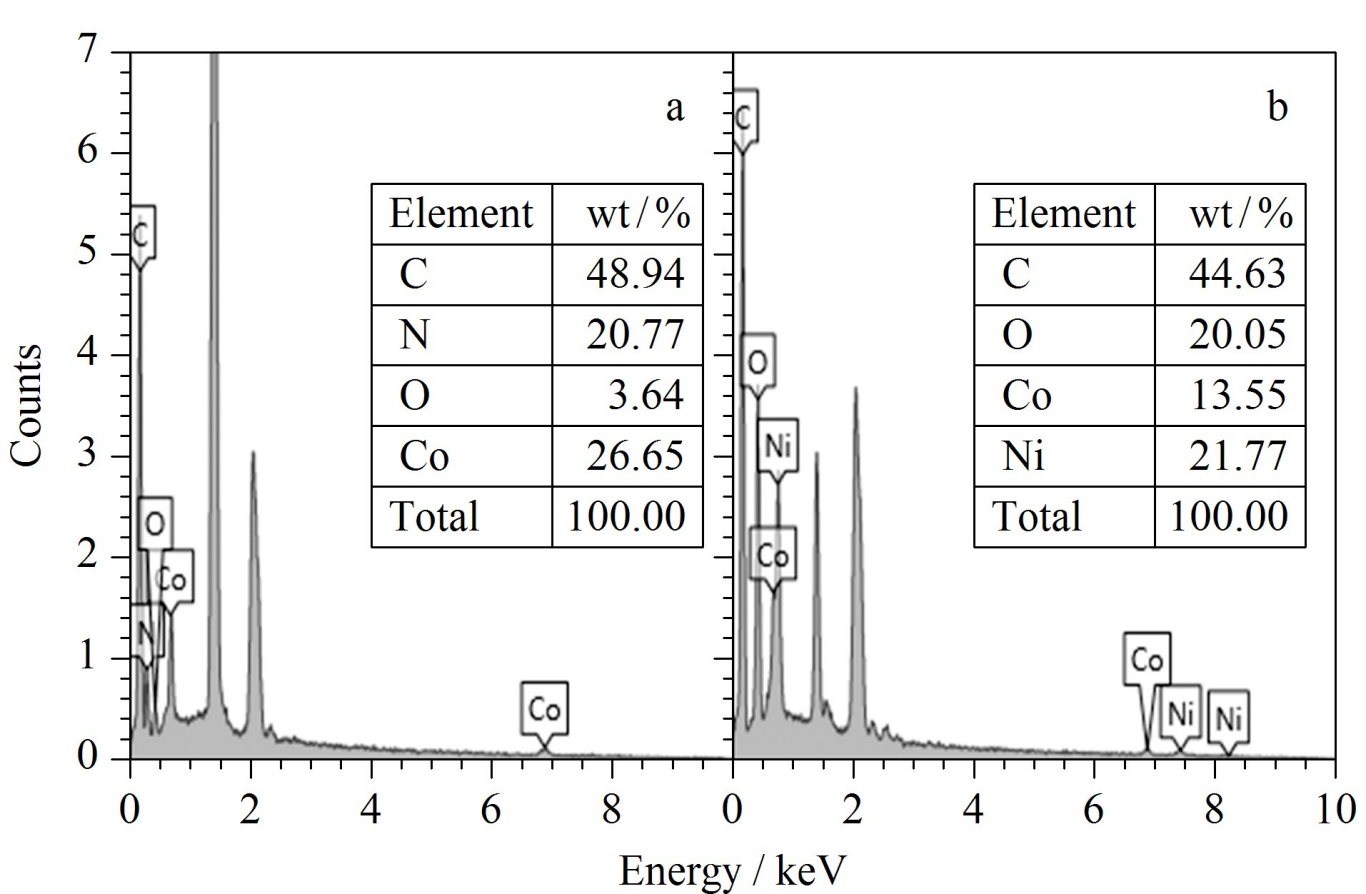
(a)ZIF-67/MWCNTs和(b)CoNi-LDH/MWCNTs的EDS光谱

#### 2.1.3 傅里叶变换红外光谱(FT-IR)

通过FT-IR进一步分析了所制备材料的不同官能团的信息及化学结构。如图4a所示,ZIF-67和ZIF-67/MWCNTs的FT-IR具有相似的吸收谱带,这证实了纳米复合材料的成功制备,其中,1421 cm^-1^和425 cm^-1^处的峰分别是C-N和Co-N振动带的特征吸收峰,1137 cm^-1^和1006 cm^-1^处的特征峰可归属于2-甲基咪唑中C=N的伸缩振动。在MWCNTs-COOH的FT-IR谱图中,2922、1732、1115、1385 cm^-1^处的吸收峰分别表示羧基中O-H、C=O、C-O的伸缩振动和O-H的弯曲振动,这结果说明了MWCNTs已经被功能化。此外,通过CoNi-LDH/MWCNTs的FT-IR谱图可以看出,3456 cm^-1^和1635 cm^-1^处的特征峰归属于O-H的伸缩振动,500~1000 cm^-1^处的峰则代表CoNi-LDH三维纳米片表面上M-O-M振动带的特征吸收峰,而1386 cm^-1^和1456 cm^-1^处的特征峰则归属于C=O的伸缩振动,这也进一步表明MWCNTs上的ZIF-67被成功地合成为CoNi-LDH三维纳米材料。

**图4:**
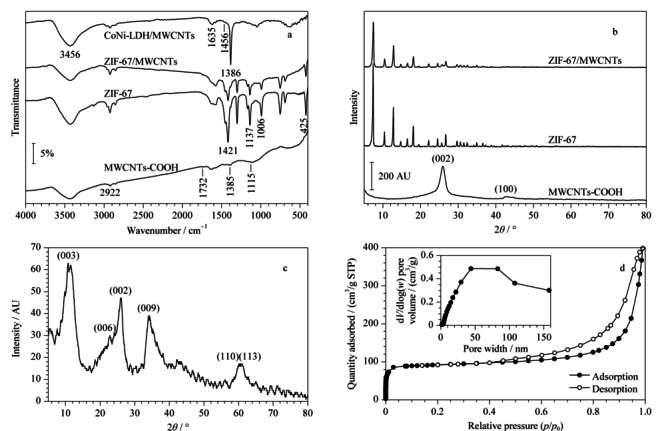
(a)MWCNTs-COOH、ZIF-67、ZIF-67/MWCNTs和CoNi-LDH/MWCNTs的红外光谱图, (b)MWCNTs-COOH、ZIF-67和ZIF-67/MWCNTs的XRD图, (c)CoNi-LDH/MWCNTs的XRD谱图和(d)CoNi-LDH/MWCNTs的N_2_吸附-脱附曲线和孔径分布图(插图)

#### 2.1.4 粉末X射线衍射(XRD)

MWCNTs-COOH、ZIF-67、ZIF-67/MWCNTs和CoNi-LDH/MWCNTs的晶相结构通过广角XRD进行分析。如图4b所示,MWCNTs-COOH在26.0°和44.2°处的特征衍射峰归因于典型的石墨(002)和(100)晶面。ZIF-67的XRD谱图与文献^[23]^的报道一致且这组峰也出现在ZIF-67/MWCNTs的XRD谱图中,这表明MWCNTs的存在并没有影响MOF晶体的形成,ZIF-67的结构没有发生变化。此外,CoNi-LDH/MWCNTs的衍射峰(见图4c)与文献^[24]^中CoNi-LDH的谱图基本相同,在10.7°(003)、22.8°(006)和33.9°(009)处的衍射峰表明其为二维层状结构,在58~60°处的(110)和(113)晶面反映了该复合材料存在两种金属阳离子。这些数据再次表明CoNi-LDH/MWCNTs复合材料已被成功地制备。

#### 2.1.5 N_2_吸附-脱附等温线

本文通过N_2_吸附-脱附等温线研究SPME的纤维涂层CoNi-LDH/MWCNTs复合材料的表面积和孔径分布。由图4d可以看出,该复合材料的N_2_吸附-脱附等温线符合第IV类吸附等温线且具有H3型滞后环,这与片状颗粒堆积形成的不均匀缝状孔隙有关。经计算得出CoNi-LDH/MWCNTs的BET表面积和孔体积分别为281.4 m^2^/g和0.49 cm^3^/g,孔径分布曲线(见图4d中的插图)在4.6~160.3 nm范围内呈多峰分布,中孔较多,这表明该复合材料的表面上存在孔洞,堆叠的纳米片(CoNi-LDH由片状颗粒堆积形成)中也存在孔隙。

### 2.2 SPME条件的优化

影响SPME效率的因素众多,且各因素之间可能存在交互作用,因此,有必要合理地运用实验设计与优化方法来进行萃取条件的筛选与优化。本实验采用五因素四水平的L_16_(4^5^)正交试验研究了几种可能影响SPME萃取性能的因素(包括萃取温度、萃取时间、搅拌速率、解吸时间和盐浓度),以获得CoNi-LDH/MWCNTs涂层纤维对6种农药的最佳萃取效率。在本实验中,使用50 μg/L的农药标准溶液优化实验参数。结果如图5和表2所示,在第11次正交设计条件下,所得萃取效果最佳,即萃取温度40 ℃、萃取时间30 min、搅拌速率500 r/min、解吸时间6 min和盐质量浓度150 mg/L为最佳的萃取条件。通过极差数据对影响萃取性能的主要因素进行了分析和评估。结果表明,对于毒死蜱、仲丁灵和哒螨灵,影响萃取性能的主要因素是萃取时间,而盐浓度影响小;对于戊唑醇和溴氰菊酯, 盐浓度影响最大,萃取温度影响最小;搅拌速率对百菌清的影响最大,萃取温度对其影响最小。产生上述结果可能是由于各目标分析物的物理化学性质不同以及CoNi-LDH/MWCNTs涂层纤维与其产生的相互作用力不同所导致的。

**图5:**
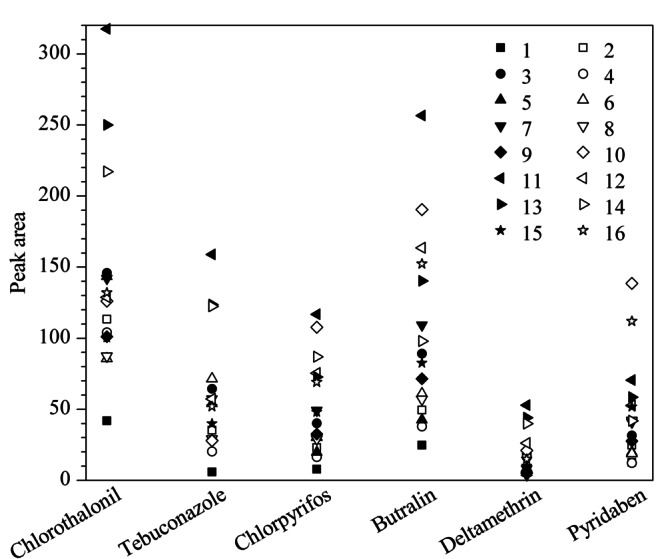
正交试验优化结果

**表2:** 五因素四水平L_16_(4^5^)正交试验-6种农药

Item			Et/min	ET/℃		Sr/(r/min)		Dt/min		ρ(NaCl)/(mg/L)		Peak areas											
												Chlorothalonil		Tebuconazole		Chlorpyrifos		Butralin		Deltamethrin			Pyridaben
1	10			20		200		4		0		41.85		5.900		7.900		24.85		8.400			12.70
2	10			30		300		6		50		113.4		35.25		23.10		49.45		5.400			24.80
3	10			40		400		8		100		145.8		64.45		40.05		89.00		18.70			31.50
4	10			50		500		10		150		104.0		20.35		16.30		38.05		16.40			12.20
5	20			20		300		8		150		143.5		54.05		19.75		42.50		8.600			19.80
6	20			30		200		10		100		85.70		71.60		30.45		61.00		5.750			18.95
7	20			40		500		4		50		141.9		57.30		49.25		109.2		8.050			40.90
8	20			50		400		6		0		87.40		29.15		29.15		57.05		3.800			42.00
9	30			20		400		10		50		101.0		28.25		32.60		71.55		5.250			27.60
10	30			30		200		8		0		126.1		28.00		107.8		190.4		21.10			138.5
11	30			40		500		6		150		317.6		159.0		116.8		256.6		52.90			70.45
12	30			50		300		4		100		128.8		57.40		75.40		163.6		26.25			52.65
13	40			20		500		6		100		250.0		123.7		72.65		140.2		44.15			58.45
14	40			30		400		4		150		217.2		122.3		86.80		97.90		39.90			41.95
15	40			40		300		10		0		100.3		39.95		47.90		82.55		10.10			51.60
16	40			50		200		8		50		132.0		51.85		69.00		152.2		15.00			112.0
I_10_	Et											101.3		31.49		21.84		50.34		12.23			20.30
I_20_												114.6		53.03		32.15		67.44		6.550			30.41
I_30_												168.4		68.16		83.15		170.5		26.38			72.30
I_40_												174.9		84.45		69.09		118.2		27.29			94.78
I_Et_												73.58		52.96		61.31		120.2		20.74			74.48
I_T20_				ET								134.1		52.98		33.23		69.78		16.60			29.64
I_T30_												135.6		64.29		62.04		99.69		18.04			56.05
I_T40_												176.4		80.18		63.50		134.3		22.44			48.61
I_T50_												113.1		39.69		47.46		102.7		15.36			54.71
I_ET_												63.35		40.49		30.28		64.56		7.075			26.41
I_200_						Sr						96.41		39.34		53.79		107.1		12.56			70.54
I_300_												121.5		46.66		41.54		84.53		12.59			37.21
I_400_												137.9		61.04		47.15		78.88		16.91			35.76
I_500_												203.4		90.09		63.75		136.0		30.38			45.50
I_Sr_												107.0		50.75		22.21		57.14		17.81			34.78
I_4_								Dt				132.4		60.73		54.84		98.89		20.65			37.05
I_6_												192.1		86.78		60.43		125.8		26.56			48.93
I_8_												136.9		49.59		59.15		118.5		15.85			75.45
I_10_												97.75		40.04		31.81		63.29		9.375			27.59
I_Dt_												94.35		46.74		28.61		62.54		17.19			47.86
I_0_										NaCl		88.91		25.75		48.19		88.71		10.85			61.20
I_5_												122.1		43.16		43.49		95.60		8.425			51.33
I_10_												152.6		79.29		54.64		113.5		23.71			40.39
I_15_												195.6		88.93		59.91		108.8		29.45			36.10
I_NaCl_												106.7		63.18		16.43		24.74		21.03			25.10

Et: extraction time; ET: extraction temperature; Sr: stirring rate; Dt: desorption time; *ρ*(NaCl): mass concentration of NaCl.

### 2.3 分析方法验证

在上述优化的萃取条件下,采用CoNi-LDH/MWCNTs涂层纤维对含有6种农药的标准溶液进行吸附,并结合HPLC-UV进行分析方法的验证。5次平行试验测得的结果如表3所示,本实验所建立的方法对农药具有宽的线性范围(百菌清为0.015~200 μg/L,戊唑醇为0.140~200 μg/L,毒死蜱为0.250~200 μg/L,仲丁灵为0.077~200 μg/L,溴氰菊酯为1.445~200 μg/L,哒螨灵为0.964~200 μg/L),且线性相关系数(*R*^2^)均大于0.9983。检出限(LOD,信噪比(*S/N*)=3)和定量限(LOQ,*S/N*=10)分别在0.004~0.434 μg/L和0.015~1.445 μg/L之间。此外,日内和日间精密度(以RSD表示)的范围分别为0.6%~5.7%和0.5%~3.4%。不同批次纤维间的重复性RSD为0.5%~4.8%。结果表明该方法具有较低的检出限,良好的精密度和可靠的重复性。

**表3:** 所建立方法的分析参数(*n*=5)

Analyte	Linear range/(μg/L)	R^2^	Precisions (RSDs/%)		Fiber-to-fiber reproducibility (RSD/%)	LOD/(μg/L)	LOQ/(μg/L)
			Intra-day	Inter-day			
Chlorothalonil	0.015-200	0.9996	0.6	0.5	2.7	0.004	0.015
Tebuconazole	0.140-200	0.9997	1.7	1.9	4.3	0.042	0.140
Chlorpyrifos	0.250-200	0.9983	1.9	1.3	1.1	0.075	0.250
Butralin	0.077-200	0.9996	1.2	0.9	2.2	0.023	0.077
Deltamethrin	1.445-200	0.9991	5.6	3.4	0.5	0.434	1.445
Pyridaben	0.964-200	0.9995	5.7	1.5	4.8	0.289	0.964

### 2.4 实际水样分析

为了评估CoNi-LDH/MWCNTs涂层纤维的实用性,将该方法应用于3个真实水样中农药的检测。结果如表4所示,在民勤河上游和下游中均检测到百菌清、戊唑醇、仲丁灵和哒螨灵,且质量浓度分别在2.789~6.425 μg/L和5.167~7.531 μg/L之间。在东大沟河检出戊唑醇、仲丁灵和哒螨灵(3.032~7.766 μg/L)。将10 μg/L和50 μg/L的农药标准溶液分别加标到3个水样中进行回收率试验,进一步评估该方法的准确性和基质影响,结果显示民勤河上游、下游和东大沟水样的加标回收率分别为87.8%~103.1%、86.6%~105.5%和83.9%~108.2%, RSD分别小于5.3%、4.2%和5.1%。这表明该方法可靠实用,适用于实际水样中痕量农药的检测。

**表4:** 实际样品中6种农药的检测结果及在两个水平下的加标回收率(*n*=3)

Sample source	Analyte	Original/(μg/L)	Spiked at 10 μg/L				Spiked at 50 μg/L		
			Found/(μg/L)	Recovery/%	RSD/%		Found/(μg/L)	Recovery/%	RSD/%
Upper reaches of the	chlorothalonil	5.015	14.97	99.55	2.67		53.56	97.09	3.14
Minqin River	tebuconazole	3.117	12.02	89.03	1.75		49.88	93.53	2.85
	chlorpyrifos	ND	10.12	101.2	4.28		44.53	89.06	5.25
	butralin	6.425	15.46	90.35	3.28		50.33	87.81	2.08
	deltamethrin	ND	9.991	99.91	1.92		44.20	88.40	4.58
	pyridaben	2.789	13.10	103.1	1.49		53.75	101.9	2.74
Lower reaches of the	chlorothalonil	7.531	17.27	97.39	3.48		54.25	93.44	1.81
Minqin River	tebuconazole	5.650	14.31	86.60	3.88		51.15	91.00	3.93
	chlorpyrifos	ND	9.856	98.56	1.08		51.12	102.2	4.12
	butralin	8.413	18.96	105.5	4.16		53.01	89.19	2.81
	deltamethrin	ND	10.17	101.7	3.36		49.30	98.60	1.97
	pyridaben	5.167	14.73	95.63	1.53		53.75	97.17	2.79
Dongdagou River	chlorothalonil	ND	9.443	94.43	2.54		51.40	102.8	3.38
	tebuconazole	3.465	12.83	93.65	1.35		53.39	99.85	3.31
	chlorpyrifos	ND	10.82	108.2	5.05		50.95	101.9	4.94
	butralin	3.032	11.65	86.18	2.79		46.85	87.64	2.42
	deltamethrin	ND	9.810	98.10	2.44		43.42	86.84	4.36
	pyridaben	7.766	16.16	83.94	2.34		53.97	92.41	4.31

ND: not detected or lower than limits of detection.

### 2.5 萃取性能的研究

为了评价CoNi-LDH/MWCNTs涂层纤维的萃取性能,选择MWCNTs-COOH、ZIF-67、ZIF-67/MWCNTs和硅密封胶等涂层纤维与其进行比较,在各自最优的SPME条件下富集相同含量(100 μg/L)的农药,经HPLC分析后得到的谱图如图6所示,结果表明CoNi-LDH/MWCNTs涂层纤维的萃取效率明显优于其他SPME纤维,这归因于它的高比表面积和优异的吸附性,此外,CoNi-LDH/MWCNTs材料与目标分析物之间存在的*π*-*π*堆积作用、疏水作用、阳离子-*π*相互作用以及氢键作用也增强了其对农药的萃取能力。

**图6:**
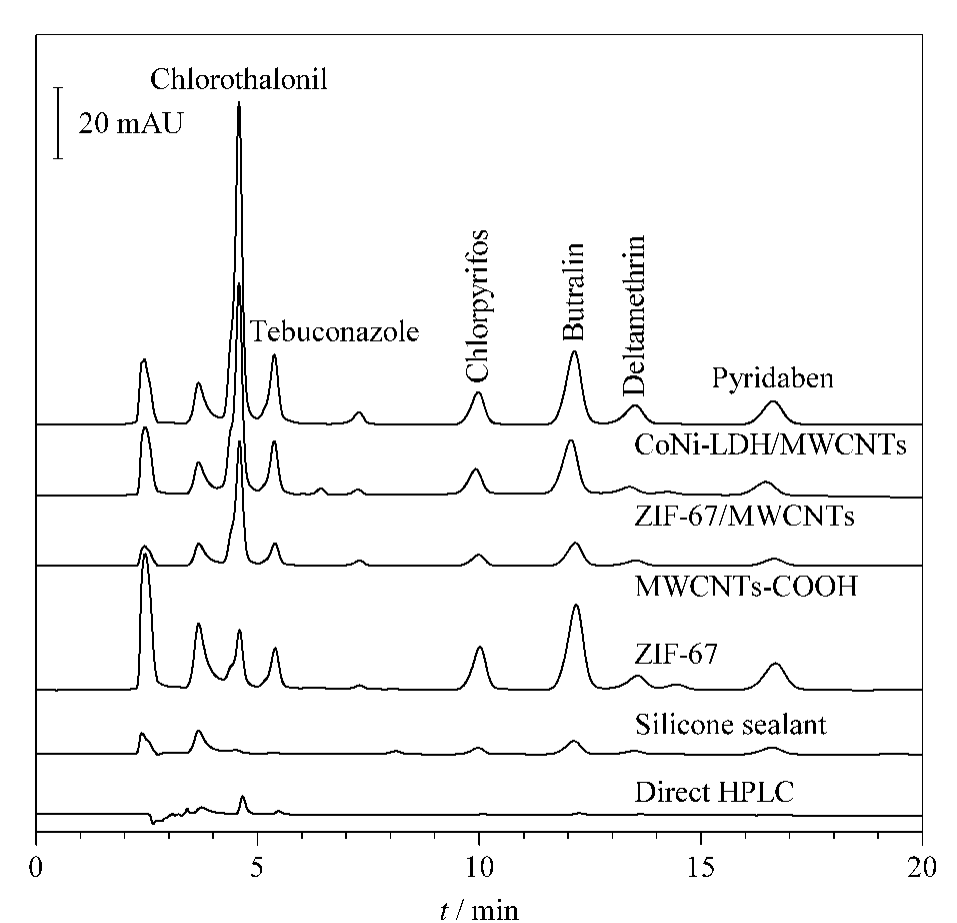
CoNi-LDH/MWCNTs涂层和其他材料涂层萃取农药后得到的色谱图

### 2.6 纤维稳定性

耐久性是评价SPME纤维涂层稳定性的重要指标。因此,为了评估CoNi-LDH/MWCNTs涂层纤维的耐久性,在该涂层纤维循环使用76次和128次之后,测定了其对6种农药(100 μg/L)的萃取效率,结果如图7所示,经过128次循环之后,CoNi-LDH/MWCNTs涂层纤维对6种农药的萃取效率只是轻微的降低(<10%),这表明CoNi-LDH/MWCNTs涂层纤维具有良好的稳定性和重复使用性。

**图7:**
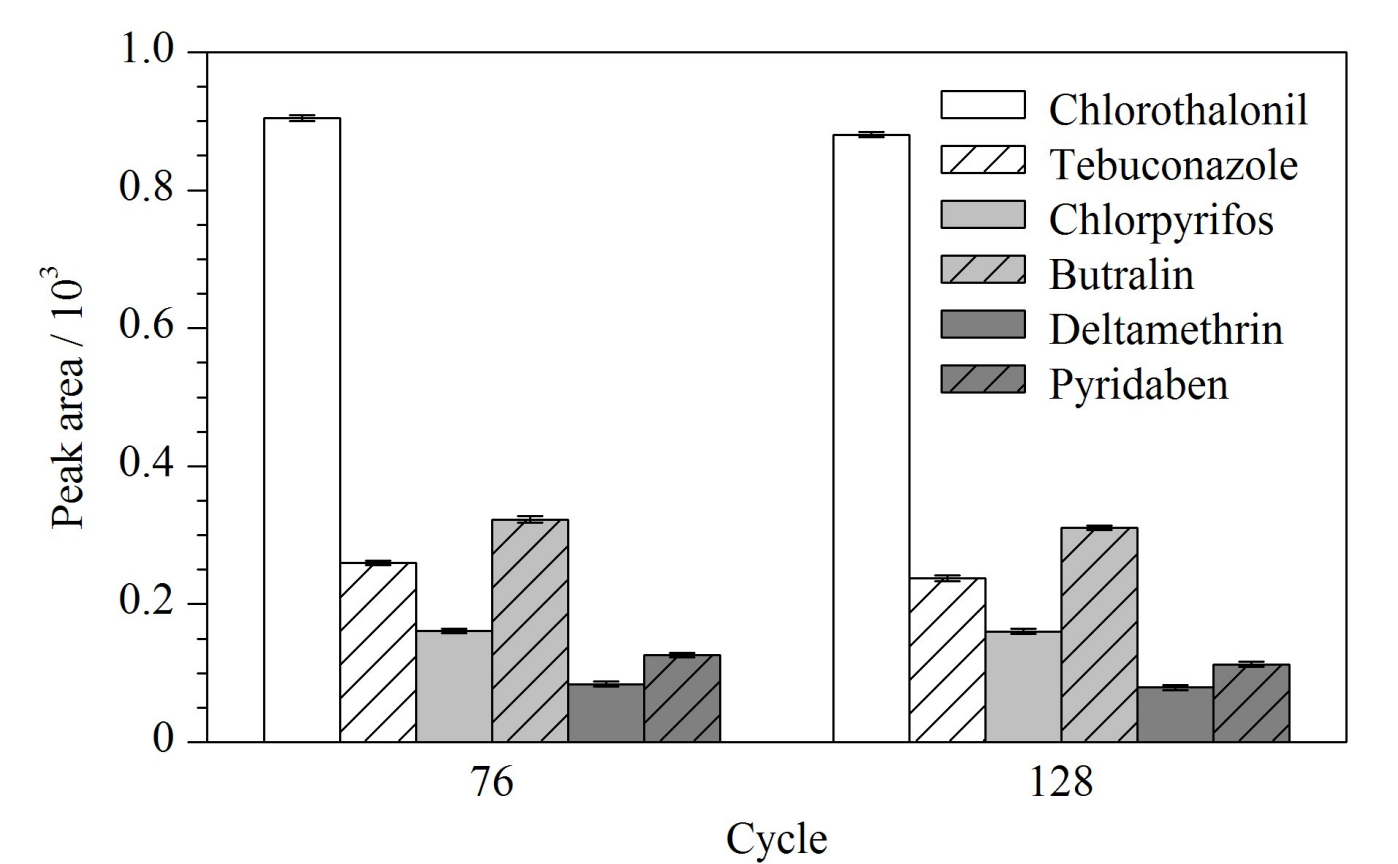
经过76次和128次循环后CoNi-LDH/MWCNTs涂层纤维对农药(100 μg/L)的萃取效果(*n*=3)

### 2.7 与其他方法的比较

为了证明该方法的优越性,将该方法与其他农药分析方法在分析技术、吸附剂类型、样品数量、提取时间、线性范围和检出限等方面进行了比较^[25⇓⇓-28]^。相关数据如表5所示,通过对比可以看出,该方法的提取时间最短,检出限最低。

**表5:** 本方法与其他文献中农药分析方法的对比

Methods	Sorbent	Analyte type	Type of analytes	Extraction time/min	Linear range/(μg/L)		LODs/(μg/L)		Ref.
DSPE-HPLC-UV	Zn-BTC MOFs	pesticides	7	30	10.0-	1000	0.20-	1.60	[25]
SBSE-HPLC-PDA	MON-2COOH	herbicides	4	40	0.10-	250	0.025-	0.070	[26]
HS-SPME-GC-FPD	G/QAS	pesticides	8	40	1-	1000	0.03-	1.01	[27]
SBSE-HPLC-DAD	PAF-56p	herbicides	6	55	0.1-	200	0.037-	0.089	[28]
SPME-HPLC-UV	CoNi-LDH/MWCNTs	pesticides	6	30	0.015-	200	0.004-	0.434	this work

DSPE: dispersive solid-phase extraction; SBSE: stir bar sorptive extraction; HS-SPME: headspace solid-phase microextraction.

## 3 结论

本实验利用MWCNTs表面生长的ZIF-67为钴源和模板制备出具有三维笼状结构的CoNi-LDH。与传统层状的双金属氢氧化物相比,其笼状结构有效地防止了LDHs在MWCNTs上聚集和堆积,加快了传质速率。另一方面,MWCNTs-COOH上含有的大量含氧官能团与农药之间存在的作用力也提高了其对农药的吸附性和富集能力。将制得的CoNi-LDH/MWCNTs作为SPME纤维的涂层,结合HPLC-UV分析环境水样中的6种农药。*π*-*π*堆积作用、疏水作用、阳离子-*π*相互作用以及氢键作用使CoNi-LDH/MWCNTs-SPME纤维比其他材料纤维具有更好的富集能力。此外,建立的CoNi-LDH/MWCNTs-SPME-HPLC-UV方法具有较宽的线性范围、较低的检出限、较高的回收率以及良好的重复性。综上所述,该方法为复杂基质中痕量有机污染物的分离富集提供了广阔的应用前景。
